# Prevalence of Dementia among Patients Hospitalized with Type 2 Diabetes Mellitus in Spain, 2011–2020: Sex-Related Disparities and Impact of the COVID-19 Pandemic

**DOI:** 10.3390/ijerph20064923

**Published:** 2023-03-10

**Authors:** Ana Lopez-de-Andres, Rodrigo Jimenez-Garcia, Jose J. Zamorano-Leon, Ricardo Omaña-Palanco, David Carabantes-Alarcon, Valentin Hernández-Barrera, Javier De Miguel-Diez, Natividad Cuadrado-Corrales

**Affiliations:** 1 Department of Public Health and Maternal & Child Health, Faculty of Medicine, Universidad Complutense de Madrid, 28040 Madrid, Spain; 2 Preventive Medicine and Public Health Teaching and Research Unit, Health Sciences Faculty, Universidad Rey Juan Carlos, 28922 Alcorcón, Spain; 3 Respiratory Care Department, Hospital General Universitario Gregorio Marañón, Instituto de Investigación Sanitaria Gregorio Marañón (IiSGM), Universidad Complutense de Madrid, 28007 Madrid, Spain

**Keywords:** type 2 diabetes, dementia, Alzheimer’s disease, vascular disease, COVID-19, in-hospital mortality, hospitalization, sex

## Abstract

(1) Background: To assess changes in the prevalence of dementia among patients hospitalized with type 2 diabetes (T2DM), to analyze the effects of dementia on in-hospital mortality (IHM) in this population, to evaluate sex differences, and to determine the impact of the COVID-19 pandemic on these parameters. (2) Methods: We used a nationwide discharge database to select all patients with T2DM aged 60 years or over admitted to Spanish hospitals from 2011 to 2020. We identified those with all-cause dementia, Alzheimer’s disease (AD), and vascular dementia (VaD). The effect of sex, age, comorbidity, and COVID-19 on the prevalence of dementia subtypes and on IHM was assessed using multivariable logistic regression. (3) Results: We identified 5,250,810 hospitalizations with T2DM. All-cause dementia was detected in 8.31%, AD in 3.00%, and VaD in 1.55%. The prevalence of all subtypes of dementia increased significantly over time. After multivariable adjustment, higher values were observed in women for all-cause dementia (OR 1.34; 95% CI 1.33–1.35), AD (OR 1.6; 95% CI 1.58–1.62), and VaD (OR 1.12; 95% CI 1.11–1.14). However, female sex was a protective factor for IHM in patients with all-cause dementia (OR 0.90; 95% CI 0.89–0.91), AD (OR 0.89; 95% CI 0.86–0.91), and VaD (OR 0.95; 95% CI 0.91–0.99). IHM among patients with dementia remained stable over time, until 2020, when it increased significantly. Higher age, greater comorbidity, and COVID-19 were associated with IHM in all dementia subtypes. (4) Conclusions: The prevalence of dementia (all-cause, AD, and VaD) in men and women with T2DM increased over time; however, the IHM remained stable until 2020, when it increased significantly, probably because of the COVID-19 pandemic. The prevalence of dementia is higher in women than in men, although female sex is a protective factor for IHM.

## 1. Introduction

Recent years have seen an increase in the incidence of neurodegenerative diseases such as dementia, partially owing to the increase in life expectancy and in the prevalence of type 2 diabetes mellitus (T2DM) [1,2].

Dementia is a highly prevalent progressive disorder, and according to the World Health Organization, it is estimated that almost 10 million new cases will appear each year [3].

Compared with persons who do not have diabetes, those with T2DM are 1.5 to 2.5 times more likely to develop all-cause dementia, including its two main subtypes, Alzheimer’s disease (AD) and vascular dementia (VaD) [4,5]. This increased risk in patients with T2DM translates into an average earlier onset of dementia of 2.5 years compared with patients without T2DM [6]. The complex pathophysiology underlying this relationship may involve hyperglycemia, insulin resistance, neuroinflammation, and altered energy homeostasis [4,5]. Given the association between the aging global population and the rising prevalence of dementia, the link between T2DM and dementia constitutes a global public health concern [1,2,7].

Diabetes is a major comorbidity among patients hospitalized with COVID-19 [8]. Dementia can increase the risk of a poorer COVID-19 outcome. In the US, a study using a nationwide database found that dementia was associated with COVID-19-related hospitalization in patients with T2DM (OR 2.07; 95% CI 1.79–2.39). However, the authors did not find dementia to be associated with in-hospital mortality (IHM) during COVID-19-related hospitalization in patients with T2DM (OR 0.85; 95% CI 0.71–1.02) [9].

Sex differences may play a critical role in the incidence and outcomes of hospitalizations in patients with T2DM and dementia. Women with T2DM have a higher excess risk of cognitive decline and vascular dementia than men with T2DM, although the extent of these differences depends on the characteristics of the study populations and the methods used [10]. In any case, it remains unclear why men and women with T2DM are affected differently by dementia [11].

Therefore, the objectives of the study were to assess changes in the prevalence of dementia (all-cause, AD, and VaD) in hospitalized patients with T2DM in Spain between 2011 and 2020. We evaluated sex differences in the prevalence, clinical characteristics, and IHM of dementia between men and women with T2DM. We also analyzed which variables were associated with IHM among patients with T2DM and dementia. Finally, we assessed whether the COVID-19 pandemic affected the prevalence of dementia and IHM in patients hospitalized with T2DM in the year 2020.

## 2. Materials and Methods

### 2.1. Design and Data Source

A retrospective, population-based observational study was conducted using the Spanish National Hospital Discharge Database (RAE-CMBD, Registro de Actividad de Atención Especializada-Conjunto Mínimo Básico de Datos [Register of Specialized Care–Basic Minimum Database]). A description of the RAE-CMBD methodology is available online [12]. The study period ran from 1 January 2011 to 31 December 2020.

The RAE-CMBD collects age, sex, dates of admission and discharge, discharge destination (home, deceased, social institution, or voluntary discharge), up to 20 diagnoses, and 20 procedures conducted during hospitalization in either public and private hospitals. The International Classification of Diseases, Ninth Revision, Clinical Modification (ICD-9-CM) was used for coding between 2011 and 2015, and the International Classification of Disease, Tenth Revision (ICD10) has been used since 2016. 

### 2.2. Study Population and Study Variables

The study population included patients aged ≥60 years with a T2DM code in any di-agnostic position (see ICD codes in Table S1). Patients with T1DM and data missing for sex, age, dates of admission and discharge, or discharge destination were excluded.

To respond to the objectives of the study, the study population was stratified according to the presence of ICD codes for dementia (all-cause dementia, AD, and VaD) in any diagnostic position in the RAE-CMBD (Table S1). All analyses were subsequently stratified according to sex.

The presence of comorbidity was assessed using the Charlson Comorbidity Index (CCI), excluding diabetes and dementia, and the ICD codes described by Sundararajan et al. [13] and Quan et al. [14]. Likewise, regardless of the diagnostic position, the presence of COVID-19 was evaluated (see ICD10 codes in Table S1) in the year 2020.

Regarding hospital outcomes, we analyzed IHM, which was defined as the number of patients who died in hospital each year divided by the total number of hospitalizations that year.

### 2.3. Statistical Analysis

We calculated the total prevalence of all-cause dementia, AD, and VaD in patients with T2DM according to year, sex, and age group.

The results of the descriptive statistical analysis are expressed as total frequencies with percentages for categorical variables and means with standard deviations for continuous variables.

The trend was analyzed using the Cochran–Mantel–Haenszel statistic or Cochran–Armitage test in the case of categorical variables and a linear regression *t* test or Jonckheere–Terpstra test in the case of continuous variables.

Categorical variables were compared using the Fisher exact test. Continuous variables were compared using the *t* test.

We used multivariable logistic regression to analyze factors associated with the presence of all-cause dementia, AD, and VaD, considering the effect of sex and the other study covariates. We also identified the variables associated with IHM in men and women with T2DM and all-cause dementia, AD, and VaD. 

We used the “enter modelling” method for logistic regression. This included five consecutive steps. First, a bivariate analysis of each variable. Second, the selection of variables for the multivariable analysis, with those with a *p* value of <0.10 being considered. Third, the contribution to the model of each variable was verified using the Wald statistic Forth, as variables were progressively included, the new model generated was compared to the previous one using the likelihood-ratio test. Finally, once the final model was obtained, we checked for linearity and possible interactions between variables. The results of these models are shown with the odds ratio (OR) and 95% confidence intervals (CI).

We used Stata version 14 to perform the statistical analysis (Stata, College Station, TX, USA). Statistical significance was set at *p* < 0.05 (2-tailed).

### 2.4. Ethics Statement

To carry out this study, it was not necessary to request the informed consent of the patients or approval by an ethics committee, since the RAE-CMBD is an administrative database, and all personal data are anonymized. Any investigator can freely request RAE-CMBD data from the Spanish Ministry of Health [15].

## 3. Results

Between 2011 and 2020 in Spain, there were 5,250,810 hospitalizations of patients aged ≥60 years presenting a diagnosis code corresponding to T2DM. Of these, 8.31% (*n* = 436,533) had an all-cause dementia code, 3.00% (*n* = 157,674) had an AD code, and 1.55% (*n* = 81,146) had a VaD code.

### 3.1. Time Trends in the Prevalence of Dementia in Patients with T2DM

The prevalence of all-cause dementia among hospitalized patients with T2DM in Spain increased significantly between 2011 and 2020 (6.9% vs. 10.6%; *p* < 0.001). Likewise, the prevalence of AD and of VaD rose significantly throughout the study period (2.71% and 1.55% in 2011 vs. 3.28% and 1.63% in 2020; all *p* < 0.001, respectively).

As can be seen in Table 1, in patients presenting with all-cause dementia, AD, and VaD, the proportion of men increased over time, while that of women decreased (all *p* < 0.001). The mean age of patients with dementia (all-cause, AD, and VaD), as well as an associated comorbidity (CCI), increased significantly over the study period.

In the three diseases under study, IHM remained stable between 2011 and 2019. However, in 2020, the IHM increased by around three percentage points, reaching 18.72% for all-cause dementia, 18.85% for AD, and 16.91% for VaD (Table 1).

### 3.2. Sex Differences in the Prevalence and Characteristics of Dementia among Patients with T2DM

As can be seen in Table 2, the prevalence of all-cause dementia was higher in women than in men with T2DM for all the study years, increasing significantly between 2011 and 2020 in both groups (4.91% to 8.4% in men; 9.19% to 19.97% in women: *p* < 0.001). 

Women with all-cause dementia were older than men, although they had a lower CCI (Table 2). Age and concomitant comorbidity increased significantly between 2011 and 2020 in both men and women. IHM in men and women with all-cause dementia increased significantly over the study period (16.03% and 14.76% in 2011 to 19.97% and 17.67% in 2020, respectively).

As shown in Table 3, the prevalence of AD increased significantly between 2011 and 2020 in both men (1.74% to 2.07%; *p* < 0.001) and women (3.82% to 4.92%; *p* < 0.001), although it was higher in women for all the years studied. The distribution by age and comorbidity shows the same trend as in T2DM patients admitted with all-cause dementia.

Between 2011 and 2020, the IHM in T2DM patients with AD increased significantly (from 15.6% to 20.35% in men and from 13.88% to 18% in women) (Table 3).

The prevalence of VaD increased throughout the study period (Table 4) and was higher in women than in men. The distribution by age, comorbidity (expressed as the mean of the CCI), and IHM shows the same pattern as that described in the previous types of dementia.

### 3.3. Variables Associated with Sex Differences in the Prevalence and Characteristics of Dementia among Patients with T2DM

Table 5 shows the results of the multivariable analysis to identify the factors associated with the presence of all-cause dementia and with IHM in men and women with T2DM and all-cause dementia. The presence of more comorbid conditions and the year of hospital admission (specifically the years 2013, 2014, and 2015 [reference year 2011]) were associated with a lower probability of presenting a code for all-cause dementia. However, the presence of all-cause dementia increased significantly between 2016 and 2020 in both men and women with T2DM. Older age and the presence of COVID-19 were also associated with a higher probability of presenting a code for all-cause dementia in both sexes. After adjusting for covariates, in the entire T2DM population, women were 1.34-fold more likely to have a code for all-cause dementia in their discharge report than men (OR 1.34; 95% CI 1.33–1.35) (Table 5). 

Regarding IHM, older age, greater comorbidity, and COVID-19 increased the risk of dying during hospitalization in men and women with T2DM and all-cause dementia (Table 5). In the entire study population, being a woman was associated with lower IHM (OR 0.90; 95% CI 0.89–0.91).

Table S2 and Table S3 show the results for trends in the presence of and factors associated with IHM for patients with AD and VaD. The prevalence of AD and VaD increased significantly with older age and COVID-19. However, the presence of comorbid conditions was associated with a lower presence of AD (OR 1.6; 95% CI 1.58–1.62) and with a higher presence of VaD (OR 0.66; 95% CI 0.65–0.66). Using the year 2011 as a reference, the presence of codes for AD increased significantly between the years 2014 and 2020, and the prevalence of VaD decreased between the years 2013 and 2018 and in the year 2020. Furthermore, women were 1.6-fold and 1.12-fold more likely to have a code for AD and VaD than men (OR 1.6; 95% CI 1.58–1.62 and OR 1.12; 95% CI 1.11–1.14, respectively).

The factors associated with all-cause dementia were also associated with an increased risk of IHM in patients hospitalized with T2DM and AD and VaD. These included older age, higher CCI, and COVID-19. As found for all-cause dementia, female sex was associated with a lower IHM for AD (OR 0.89; 95% CI 0.86–0.91) and VaD (OR 0.95; 95% CI 0.91–0.99).

## 4. Discussion

The results obtained in this nationwide retrospective study of over 5 million patients with T2DM aged ≥60 years admitted to Spanish hospitals between 2011 and 2020 revealed several key findings. First, an increase in the prevalence of dementia (all-cause, AD, and VaD) was observed in men and women with T2DM between 2011 and 2020. Second, the prevalence of all-cause dementia was 1.34 times higher in women than in men with T2DM. Third, the presence of COVID-19 increased the risk of IHM in men and women with T2DM and any dementia subtype. Finally, we found that women with T2DM and all-cause dementia, AD, and VaD had a lower risk of dying in hospital than T2DM men.

Several epidemiological studies report an increase in the prevalence of dementia in individuals with T2DM over time [16,17,18]. Our results confirm this trend, which has been reported elsewhere, both in AD and in VaD [17,18].

In the United Kingdom general population, using data from over 13 million individuals aged ≥18 years receiving primary care and recorded in the Health Improvement Network database, the overall prevalence of dementia among people with diabetes increased from 0.42% in year 2000 to 2.51 % in 2016 (*p* < 0.001). The prevalence of dementia in women patients with diabetes was approximately 1.5 times higher compared to men patients with diabetes [16].

A study of persons aged 90 or over with dementia in Finland showed that the prevalence of diabetes doubled between 2000 and 2018 (*p* < 0.001) [19].

The increase in the prevalence of dementia in people with diabetes and vice versa over time, can be explained by greater survival, life expectancy, and improvements in the diagnoses of both pathologies [16,17,18,19].

As expected, we found that between 2011 and 2020, both patient age and the proportion of men with TDM2 and dementia increased. In addition, patients presented greater comorbidity over time. Different studies have described an increase in the prevalence of the main age-related chronic conditions in the general elderly population [20,21,22,23,24]. Furthermore, a recent population-based registry-based study on comorbidity trends during the last years of life in Finnish patients with dementia aged 70 years or older found an increase in comorbidities between 2001 and 2013 [25]. Another cohort study of 245,483 participants showed that older adults with multiple comorbid conditions had a higher risk of dementia [26]. Other factors reported as being relevant include aging, polypharmacy, and a heavier treatment burden, all of which might affect the brain and cause neural injuries [27,28].

The prevalence of dementia is higher in women with diabetes than in men, in terms of all-cause dementia, AD, and VaD [10,11]. A recent meta-analysis found that women with diabetes had a 19% higher risk for VaD than men with diabetes (RR 1.19; 95% CI 1.08–1.30) [11]. Various factors seem to contribute to the difference between men and women regarding dementia. In the case of diabetes, it has been reported that women with T2DM achieve glycemic and cardiovascular targets less frequently and are screened less frequently for the complications of diabetes. In the case of dementia, women are generally referred later than men and experience delays in receiving adequate supportive care when they have cognitive impairment [29]. Alternative explanations include hormonal aspects. Exposure to endogenous estradiol in females has been reported to increase the risk of dementia, especially in the presence of diabetes [30]. Moreover, since the female patients in this study were post-menopausal (age ≥60 years), alterations in sex hormones could play a role [31].

Gong et al. reported that mental health symptoms (depression and anxiety) and higher waist circumference have been found associated with a greater risk of dementia in women with T2DM in comparison with men [32]. A plausible explanation for this finding is that women are more likely to be prescribed with pharmacological treatments for depression, and the use of antidepressants has been linked to a greater risk of dementia. Whether the different body composition and fat distribution observed in women and men with diabetes, partially driven by the influence of sex hormones on visceral obesity, can explain the sex differences in obesity and dementia, requires further investigation [32].

Studies conducted in the general population have suggested that factors such as blood pressure, physical activity, longer education, and former alcohol use have a different effect in men and women on the risk of developing dementia [33,34,35,36].

It is possible that combinations or patterns of risk factors explain sex differences in cognitive decline and the subsequent risk of dementia. Therefore, a multi-domain approach to understanding and analyzing risk factors is arguably the best approach for investigating sex differences in dementia risk. Broad domains of risk factors have been previously classified as biomarkers, demographic variables, lifestyle factors, medical conditions and medications, and environmental factors. A broader understanding of overall patterns of risk factors for cardiometabolic disease and neurodegeneration is needed to inform tailored (potentially sex-specific) interventions [37].

Future investigations should use methods such as Bayesian Mindsponge Framework analytics, to provide a more in-depth analysis of sex differences in the association between diabetes and dementia [38].

Previous studies have reported an increased risk of IHM in patients admitted to hospital with a diagnosis of diabetes and dementia, because this population has survived ischemic heart disease and stroke [39]. Our study showed that among men and women hospitalized with TD2M who had any type of dementia, IHM remained stable over time until the year 2020, when it showed a significant increase, probably related to SARS-CoV-2 infection.

As we expected, among hospitalized women and men with T2DM and all-cause dementia, AD, and VaD, the presence of COVID-19 was associated with IHM, as were advanced age and associated comorbidity. In a previous study conducted by our group, dementia was associated with IHM in men and women with T2DM hospitalized with COVID-19 in Spain in 2020 (OR in men, 2.42 [95% CI 2.14–2.74]; and OR in women, 1.64 [95% CI 1.46–1.84]) [40]. Previous evidence has shown that patients with dementia, especially those with comorbidities such as diabetes, are particularly vulnerable to SARS-CoV-2 infection and are more likely to develop severe illness [41].

In our study, women with diabetes hospitalized with all-cause dementia, VaD, and AD, had a lower risk of IHM than men with diabetes. This finding is consistent with previous research, which found that male sex predicts mortality in patients with dementia [42,43,44]. Connors et al. [44] found female sex to be a protective factor for mortality in patients with dementia (HR, hazard ratio 0.57; 95% CI 0.43–0.74). To our knowledge no previous investigation has analyzed the possible factors that explain the higher IHM among men than women with diabetes and concomitant dementia.

The observed increment in the prevalence of dementia among people hospitalized with diabetes found in our investigation has several practical implications. It is expected that the improvements in rates of chronic complications and longevity in diabetes patients will lead to more people with diabetes surviving into old age and developing dementia, therefore making it necessary that effective interventions are implemented [45,46].

According to the latest version of the Lancet Commission on Dementia Prevention, based on results from large cohort studies, up to 40% of dementia cases could be prevented by modifying twelve risk factors: low education, midlife hearing loss, obesity, hypertension, late-life depression, smoking, physical inactivity, diabetes, social isolation, excessive alcohol consumption, traumatic brain injury, and air pollution [47].

Population-based approaches are likely to be the most impactful, cost-effective, and meaningful to reduce the global burden of dementia. Public health campaigns are needed to raise awareness about the link between diabetes, and the other mentioned risk factors, with dementia. Campaigns must encourage lifestyle changes linked to diet, exercise, and weight loss, which could reduce the incidence of diabetes and mitigate dementia risk [47,48]. Specific interventions that could reduce the risk or slow down the progression of dementia in people with diabetes include optimizing the treatment of cardiovascular risk factors, promoting the use of statins and oral hypoglycemic agents, and a tight control of blood glucose levels with an HbA1 under 7% [49,50].

Our results provide policy makers with objective data on the burden that dementia causes among people with diabetes and very especially among women. Soto-Gordoa et al. have predicted that in Spain the number of cases of dementia will triple by 2050 unless effective interventions are implemented [51]. These is a very serious threat to the Spanish social and health care systems, as the associated economic burden will become barely sustainable. According to these authors, an intervention leading to a 20% change in risk and protective factors would reduce dementia by 9%, prevent over 100,000 cases, and save nearly EUR 4900 million in 2050 [51].

In Germany, Fink et al. estimated that a relative reduction of diabetes incidence by 1% annually would decrease dementia cases by around 30,000 [52].

As commented before, the reduction of the burden of dementia can only be achieved with multiple interventions that, taken separately, would yield only modest results. This evidence supports the need to include primary prevention in the form of reducing risk factors for both dementia and diabetes as a top priority of health policies [47,48,51,52].

The strengths of our study are the use of a national population database (RAE-CMBD), over a 10-year period, with a methodology that has been reported elsewhere [17,18]. However, our study is also subject to a series of limitations. While the RAE-CMBD collects practically all hospitalizations in Spain, it is an administrative database and does not collect all the variables included in the clinical history. Therefore, we have no data on disease severity, glycemic control, disease duration, or medication for diabetes or dementia. In addition, it was only possible to assess IHM, since we did not have information on the patients once they were discharged. However, the use of hospital discharge records and administrative databases for the diagnosis of psychiatric illnesses, including dementia, has been shown to be sufficiently sensitive and specific for epidemiological investigations [53,54]. Finally, the validity of diabetes ICD codes in health administrative databases, compared to clinical records, has been evaluated previously, concluding that it is reliable and can be used to address important research questions [55,56,57,58].

## 5. Conclusions

In conclusion, the prevalence of dementia, including the prevalence of VaD and AD, in men and women with T2DM increased between 2011 and 2020. Our data highlight important sex differences, indicating that the prevalence of all-cause dementia is 1.34 times higher in women than in men, with similar values for VaD and AD. However, female sex is a protective factor for IHM in hospitalized patients with all-cause dementia, VaD, and AD. A diagnosis of COVID-19, associated comorbidity, older age, and having been hospitalized in 2020 were predictors of IHM in men and women with T2DM and dementia (all-cause dementia, VaD, and AD). Clinicians should pay attention to the relationship between T2DM and dementia in order to avoid worse outcomes and reduce the burden of both diseases.

## Figures and Tables

**Table 1:** Characteristics of the patients hospitalized with type 2 diabetes and suffering from concomitant all-cause dementia, Alzheimer’s disease and, vascular dementia according to year (2011–2020).

	2011	2012	2013	2014	2015	2016	2017	2018	2019	2020	*p*-Value Trend
All-cause dementia, n (prevalence)	32,512 (6.9)	34,066 (7.02)	33,147 (6.67)	34,617 (6.74)	36,849 (6.92)	44,566 (8.94)	52,005 (9.45)	55,008 (9.5)	57,354 (9.7)	56,409 (10.6)	<0.001
Men, n (%)	12,382 (38.08)	12,904 (37.88)	12,854 (38.78)	13,535 (39.1)	14,455 (39.23)	18,969 (42.56)	22,561 (43.38)	24,275 (44.13)	25,337 (44.18)	25,754 (45.66)	<0.001
Women, n(%)	20,130 (61.92)	21,162 (62.12)	20,293 (61.22)	21,082 (60.9)	22,394 (60.77)	25,597 (57.44)	29,444 (56.62)	30,733 (55.87)	32,017 (55.82)	30,655 (54.34)	<0.001
Age, mean (SD)	82.65 (6.62)	82.79 (6.58)	82.9 (6.58)	83.02 (6.58)	83.28 (6.65)	83.08 (6.93)	83.29 (7)	83.36 (7.07)	83.42 (7.19)	83.24 (7.29)	<0.001
CCI, mean (SD)	0.91 (0.92)	0.92 (0.93)	0.95 (0.94)	0.94 (0.93)	0.94 (0.94)	1.09 (1.02)	1.12 (1.02)	1.14 (1.04)	1.19 (1.05)	1.16 (1.05)	<0.001
COVID-19, n(%)	0 (0)	0 (0)	0 (0)	0 (0)	0 (0)	1 (0)	2 (0)	3 (0.01)	5 (0.01)	6885 (12.21)	<0.001
IHM, n (%)	4957 (15.25)	5195 (15.25)	4842 (14.61)	4975 (14.37)	5591 (15.17)	6599 (14.81)	8027 (15.44)	8615 (15.66)	8960 (15.62)	10,560 (18.72)	<0.001
Alzheimer’s disease, n (prevalence)	12,759 (2.71)	13,698 (2.82)	13,469 (2.71)	14,603 (2.85)	15,621 (2.93)	15,486 (3.11)	17,484 (3.18)	18,472 (3.19)	18,648 (3.15)	17,434 (3.28)	<0.001
Men, n (%)	4379 (34.32)	4560 (33.29)	4719 (35.04)	5171 (35.41)	5466 (34.99)	5405 (34.9)	6269 (35.86)	6782 (36.72)	6785 (36.38)	6350 (36.42)	<0.001
Women, n (%)	8380 (65.68)	9138 (66.71)	8750 (64.96)	9432 (64.59)	10,155 (65.01)	10,081 (65.1)	11,215 (64.14)	11,690 (63.28)	11,863 (63.62)	11,084 (63.58)	<0.001
Age, mean (SD)	82.42 (6.06)	82.61 (5.95)	82.82 (6.02)	83 (6.01)	83.3 (6.07)	83.56 (6.09)	83.73 (6.05)	83.94 (6.07)	83.95 (6.15)	83.96 (6.21)	<0.001
CCI, mean (SD)	0.74 (0.84)	0.77 (0.87)	0.81 (0.87)	0.8 (0.87)	0.79 (0.87)	0.89 (0.93)	0.9 (0.93)	0.93 (0.94)	0.97 (0.97)	0.93 (0.97)	<0.001
COVID-19, n (%)	0 (0)	0 (0)	0 (0)	0 (0)	0 (0)	0 (0)	0 (0)	0 (0)	2 (0.01)	2091 (11.99)	<0.001
IHM, n (%)	1846 (14.47)	2002 (14.62)	1872 (13.9)	2028 (13.89)	2364 (15.13)	2224 (14.36)	2646 (15.13)	2882 (15.6)	2886 (15.48)	3287 (18.85)	<0.001
Vascular dementia, n (prevalence)	7313 (1.55)	7579 (1.56)	7479 (1.51)	7732 (1.51)	7935 (1.49)	7514 (1.51)	8645 (1.57)	8725 (1.51)	9562 (1.62)	8662 (1.63)	<0.001
Men, n (%)	3359 (45.93)	3617 (47.72)	3471 (46.41)	3684 (47.65)	3794 (47.81)	3557 (47.34)	4134 (47.82)	4269 (48.93)	4637 (48.49)	4313 (49.79)	<0.001
Women, n (%)	3954 (54.07)	3962 (52.28)	4008 (53.59)	4048 (52.35)	4141 (52.19)	3957 (52.66)	4511 (52.18)	4456 (51.07)	4925 (51.51)	4349 (50.21)	<0.001
Age, mean (SD)	81.73 (6.92)	81.75 (6.86)	82.23 (6.78)	82.23 (6.84)	82.52 (6.99)	82.64 (6.85)	83.09 (6.79)	83.13 (6.86)	83.07 (6.98)	83.06 (7.14)	<0.001
CCI, mean (SD)	1.28 (0.97)	1.28 (0.98)	1.32 (0.99)	1.31 (0.97)	1.3 (0.99)	1.38 (1.06)	1.41 (1.07)	1.4 (1.07)	1.43 (1.08)	1.42 (1.08)	<0.001
COVID-19, n (%)	0 (0)	0 (0)	0 (0)	0 (0)	0 (0)	0 (0)	1 (0.01)	0 (0)	0 (0)	897 (10.36)	0 (0)
IHM, n (%)	1044 (14.28)	1075 (14.18)	1032 (13.8)	1003 (12.97)	1143 (14.4)	1052 (14)	1271 (14.7)	1334 (15.29)	1350 (14.12)	1465 (16.91)	<0.001

CCI: Charlson Comorbidity Index. IHM: In-hospital mortality.

**Table 2:** Prevalence of all-cause dementia. Distribution by age and clinical characteristics and in-hospital outcomes among men and women hospitalized with type 2 diabetes in Spain, 2011–2020.

		2011	2012	2013	2014	2015	2016	2017	2018	2019	2020	*p*-Value Trend
Men	N, prevalence	12,382 (4.91)	12,904 (4.94)	12,854 (4.76)	13,535 (4.82)	14,455 (4.93)	18,969 (6.85)	22,561 (7.32)	24,275 (7.39)	25,337 (7.51)	25,754 (8.4)	<0.001
	Age, mean (SD)	80.93 (6.74)	81.08 (6.68)	81.17 (6.68)	81.35 (6.76)	81.56 (6.84)	81.41 (7.18)	81.63 (7.26)	81.69 (7.3)	81.69 (7.43)	81.53 (7.5)	<0.001
	60–69 years, n (%)	775 (6.26)	792 (6.14)	760 (5.91)	820 (6.06)	844 (5.84)	1319 (6.95)	1557 (6.9)	1627 (6.7)	1694 (6.69)	1865 (7.24)	<0.001
	70–79 years, n (%)	4025 (32.51)	4083 (31.64)	3991 (31.05)	3945 (29.15)	4051 (28.02)	5288 (27.88)	6058 (26.85)	6550 (26.98)	7345 (28.99)	7607 (29.54)	<0.001
	≥80 years, n (%)	7582 (61.23)	8029 (62.22)	8103 (63.04)	8770 (64.79)	9560 (66.14)	12,362 (65.17)	14,946 (66.25)	16,098 (66.32)	16,298 (64.32)	16,282 (63.22)	<0.001
	CCI, mean (SD)	1.09 (0.98)	1.1 (0.98)	1.12 (0.99)	1.1 (0.98)	1.11 (0.99)	1.3 (1.09)	1.31 (1.08)	1.33 (1.1)	1.38 (1.11)	1.35 (1.11)	<0.001
	COVID-19, n (%)	0 (0)	0 (0)	0 (0)	0 (0)	0 (0)	0 (0)	0 (0)	0 (0)	2 (0.01)	3360 (13.05)	<0.001
	IHM, n (%)	1985 (16.03)	2015 (15.62)	1992 (15.5)	1996 (14.75)	2278 (15.76)	3000 (15.82)	3671 (16.27)	3949 (16.27)	4154 (16.39)	5142 (19.97)	<0.001
Women	N, prevalence	20,130 (9.19)	21,162 (9.46)	20,293 (8.96)	21,082 (9.07)	22,394 (9.36)	25,597 (11.57)	29,444 (12.16)	30,733 (12.28)	32,017 (12.61)	30,655 (13.59)	<0.001
	Age, mean (SD)	83.7 (6.31)	83.83 (6.3)	84 (6.27)	84.08 (6.24)	84.38 (6.28)	84.31 (6.46)	84.56 (6.51)	84.68 (6.59)	84.79 (6.68)	84.68 (6.78)	<0.001
	60–69 years, n (%)	439 (2.18)	471 (2.23)	452 (2.23)	474 (2.25)	494 (2.21)	660 (2.58)	746 (2.53)	768 (2.5)	813 (2.54)	825 (2.69)	<0.001
	70–79 years, n (%)	4354 (21.63)	4422 (20.9)	3967 (19.55)	3888 (18.44)	3919 (17.5)	4500 (17.58)	4829 (16.4)	5110 (16.63)	5464 (17.07)	5525 (18.02)	<0.001
	≥80 years, n (%)	15,337 (76.19)	16,269 (76.88)	15,874 (78.22)	16,720 (79.31)	17,981 (80.29)	20,437 (79.84)	23,869 (81.07)	24,855 (80.87)	25,740 (80.39)	24,305 (79.29)	<0.001
	CCI, mean (SD)	0.79 (0.86)	0.81 (0.87)	0.85 (0.88)	0.84 (0.88)	0.82 (0.88)	0.93 (0.94)	0.97 (0.95)	0.98 (0.96)	1.03 (0.98)	1.01 (0.97)	<0.001
	COVID-19, n (%)	0 (0)	0 (0)	0 (0)	0 (0)	0 (0)	1 (0)	2 (0.01)	3 (0.01)	3 (0.01)	3525 (11.5)	<0.001
	IHM, n (%)	2972 (14.76)	3180 (15.03)	2850 (14.04)	2979 (14.13)	3313 (14.79)	3599 (14.06)	4356 (14.79)	4666 (15.18)	4806 (15.01)	5418 (17.67)	<0.001

CCI: Charlson Comorbidity Index. IHM: In-hospital mortality.

**Table 3:** Prevalence of Alzheimer disease. Distribution by age and clinical characteristics and in-hospital outcomes among men and women hospitalized with type 2 diabetes in Spain, 2011–2020.

		2011	2012	2013	2014	2015	2016	2017	2018	2019	2020	*p*-Value Trend
Men	N, prevalence	4379 (1.74)	4560 (1.74)	4719 (1.75)	5171 (1.84)	5466 (1.86)	5405 (1.95)	6269 (2.03)	6782 (2.06)	6785 (2.01)	6350 (2.07)	<0.001
	Age, mean (SD)	80.9 (6.22)	81.2 (5.94)	81.44 (6.12)	81.69 (6.25)	81.88 (6.2)	82.38 (6.22)	82.43 (6.17)	82.75 (6.24)	82.65 (6.28)	82.62 (6.36)	<0.001
	60–69 years, n (%)	229 (5.23)	192 (4.21)	196 (4.15)	228 (4.41)	210 (3.84)	187 (3.46)	219 (3.49)	199 (2.93)	212 (3.12)	204 (3.21)	<0.001
	70–79 years, n (%)	1439 (32.86)	1455 (31.91)	1432 (30.35)	1462 (28.27)	1509 (27.61)	1333 (24.66)	1557 (24.84)	1650 (24.33)	1757 (25.9)	1689 (26.6)	<0.001
	≥80 years, n (%)	2711 (61.91)	2913 (63.88)	3091 (65.5)	3481 (67.32)	3747 (68.55)	3885 (71.88)	4493 (71.67)	4933 (72.74)	4816 (70.98)	4457 (70.19)	<0.001
	CCI, mean (SD)	0.9 (0.9)	0.93 (0.92)	0.95 (0.92)	0.95 (0.92)	0.94 (0.92)	1.06 (1)	1.06 (0.99)	1.08 (1)	1.12 (1.03)	1.09 (1.03)	<0.001
	COVID-19, n (%)	0 (0)	0 (0)	0 (0)	0 (0)	0 (0)	0 (0)	0 (0)	0 (0)	0 (0)	768 (12.09)	<0.001
	IHM, n (%)	683 (15.6)	681 (14.93)	724 (15.34)	770 (14.89)	897 (16.41)	832 (15.39)	974 (15.54)	1101 (16.23)	1111 (16.37)	1292 (20.35)	<0.001
Women	N, prevalence	8380 (3.82)	9138 (4.09)	8750 (3.86)	9432 (4.06)	10,155 (4.25)	10,081 (4.56)	11,215 (4.63)	11,690 (4.67)	11,863 (4.67)	11,084 (4.92)	<0.001
	Age, mean (SD)	83.22 (5.82)	83.31 (5.84)	83.57 (5.83)	83.71 (5.75)	84.06 (5.86)	84.19 (5.92)	84.46 (5.85)	84.64 (5.86)	84.69 (5.94)	84.73 (5.98)	<0.001
	60–69 years, n (%)	149 (1.78)	170 (1.86)	159 (1.82)	167 (1.77)	195 (1.92)	187 (1.85)	174 (1.55)	154 (1.32)	167 (1.41)	160 (1.44)	<0.001
	70–79 years, n (%)	1936 (23.1)	2066 (22.61)	1817 (20.77)	1834 (19.44)	1785 (17.58)	1751 (17.37)	1780 (15.87)	1879 (16.07)	1950 (16.44)	1884 (17)	<0.001
	≥80 years, n (%)	6295 (75.12)	6902 (75.53)	6774 (77.42)	7431 (78.78)	8175 (80.5)	8143 (80.78)	9261 (82.58)	9657 (82.61)	9746 (82.15)	9040 (81.56)	<0.001
	CCI, mean (SD)	0.65 (0.79)	0.69 (0.84)	0.73 (0.83)	0.72 (0.83)	0.71 (0.83)	0.79 (0.88)	0.81 (0.89)	0.84 (0.9)	0.88 (0.92)	0.84 (0.92)	<0.001
	COVID-19, n (%)	0 (0)	0 (0)	0 (0)	0 (0)	0 (0)	0 (0)	0 (0)	0 (0)	2 (0.02)	1323 (11.94)	<0.001
	IHM, n (%)	1163 (13.88)	1321 (14.46)	1148 (13.12)	1258 (13.34)	1467 (14.45)	1392 (13.81)	1672 (14.91)	1781 (15.24)	1775 (14.96)	1995 (18)	<0.001

CCI: Charlson Comorbidity Index. IHM: In-hospital mortality.

**Table 4:** Prevalence of vascular dementia. Distribution by age and clinical characteristics and in-hospital outcomes among men and women hospitalized with type 2 diabetes in Spain, 2011–2020.

		2011	2012	2013	2014	2015	2016	2017	2018	2019	2020	*p*-Value Trend
Men	N, prevalence	3359 (1.33)	3617 (1.38)	3471 (1.28)	3684 (1.31)	3794 (1.29)	3557 (1.28)	4134 (1.34)	4269 (1.3)	4637 (1.37)	4313 (1.41)	<0.001
	Age, mean (SD)	79.99 (6.97)	80.16 (7.01)	80.55 (6.86)	80.65 (6.98)	80.72 (7.18)	80.92 (6.98)	81.41 (6.94)	81.53 (7.01)	81.38 (7.1)	81.31 (7.28)	<0.001
	60–69 years, n (%)	302 (8.99)	304 (8.4)	255 (7.35)	294 (7.98)	325 (8.57)	260 (7.31)	257 (6.22)	261 (6.11)	282 (6.08)	308 (7.14)	<0.001
	70–79 years, n (%)	1177 (35.04)	1247 (34.48)	1163 (33.51)	1151 (31.24)	1133 (29.86)	1060 (29.8)	1177 (28.47)	1192 (27.92)	1476 (31.83)	1338 (31.02)	<0.001
	≥80 years, n (%)	1880 (55.97)	2066 (57.12)	2053 (59.15)	2239 (60.78)	2336 (61.57)	2237 (62.89)	2700 (65.31)	2816 (65.96)	2879 (62.09)	2667 (61.84)	<0.001
	CCI, mean (SD)	1.42 (1.01)	1.41 (1)	1.45 (1.02)	1.41 (1)	1.44 (1.03)	1.55 (1.09)	1.55 (1.11)	1.53 (1.11)	1.57 (1.12)	1.57 (1.14)	<0.001
	COVID-19, n (%)	0 (0)	0 (0)	0 (0)	0 (0)	0 (0)	0 (0)	0 (0)	0 (0)	0 (0)	447 (10.36)	<0.001
	IHM, n (%)	502 (14.94)	506 (13.99)	484 (13.94)	487 (13.22)	568 (14.97)	512 (14.39)	624 (15.09)	616 (14.43)	651 (14.04)	742 (17.2)	<0.001
Women	N, prevalence	3954 (1.8)	3962 (1.77)	4008 (1.77)	4048 (1.74)	4141 (1.73)	3957 (1.79)	4511 (1.86)	4456 (1.78)	4925 (1.94)	4349 (1.93)	<0.001
	Age, mean (SD)	83.21 (6.52)	83.2 (6.39)	83.68 (6.36)	83.66 (6.39)	84.17 (6.38)	84.19 (6.35)	84.62 (6.27)	84.65 (6.35)	84.66 (6.47)	84.8 (6.55)	<0.001
	60–69 years, n (%)	113 (2.86)	110 (2.78)	109 (2.72)	109 (2.69)	96 (2.32)	87 (2.2)	96 (2.13)	90 (2.02)	124 (2.52)	106 (2.44)	<0.001
	70–79 years, n (%)	953 (24.1)	923 (23.3)	820 (20.46)	815 (20.13)	784 (18.93)	725 (18.32)	730 (16.18)	746 (16.74)	830 (16.85)	736 (16.92)	<0.001
	≥80 years, n (%)	2888 (73.04)	2929 (73.93)	3079 (76.82)	3124 (77.17)	3261 (78.75)	3145 (79.48)	3685 (81.69)	3620 (81.24)	3971 (80.63)	3507 (80.64)	<0.001
	CCI, mean (SD)	1.16 (0.92)	1.16 (0.94)	1.21 (0.95)	1.22 (0.92)	1.17 (0.93)	1.23 (0.99)	1.27 (1.02)	1.28 (1.01)	1.29 (1.01)	1.28 (1.01)	<0.001
	COVID-19, n (%)	0 (0)	0 (0)	0 (0)	0 (0)	0 (0)	0 (0)	1 (0.02)	0 (0)	0 (0)	450 (10.35)	<0.001
	IHM, n (%)	542 (13.71)	569 (14.36)	548 (13.67)	516 (12.75)	575 (13.89)	540 (13.65)	647 (14.34)	718 (16.11)	699 (14.19)	723 (16.62)	<0.001

CCI: Charlson Comorbidity Index. IHM: In-hospital mortality.

**Table 5:** Multivariable analysis of the factors associated with the presence of all-cause dementia among men and women hospitalized with type 2 diabetes and factors associated with in-hospital mortality among patients with type 2 diabetes and concomitant all-cause dementia in Spain, 2011–2020.

	Presence of All-Cause Dementia			IHM of Patients with T2DM and All-Cause Dementia		
	Men	Women	Both Sexes	Men	Women	Both Sexes
	OR (95% CI)	OR (95% CI)	OR (95% CI)	OR (95% CI)	OR (95% CI)	OR (95% CI)
Year 2011	1	1	1	1	1	1
Year 2012	0.99 (0.96–1.01)	1.02 (1–1.04)	1.01 (0.99–1.02)	0.97 (0.9–1.03)	1.02 (0.96–1.07)	1 (0.95–1.04)
Year 2013	0.95 (0.92–0.97)	0.96 (0.94–0.98)	0.95 (0.94–0.97)	0.95 (0.89–1.02)	0.93 (0.88–0.98)	0.94 (0.9–1.01)
Year 2014	0.95 (0.92–0.97)	0.96 (0.94–0.98)	0.95 (0.94–0.97)	0.89 (0.84–0.96)	0.93 (0.88–0.98)	0.92 (0.88–1.00)
Year 2015	0.95 (0.93–0.97)	0.97 (0.95–0.99)	0.96 (0.94–0.97)	0.96 (0.9–1.03)	0.98 (0.93–1.04)	0.97 (0.93–1.01)
Year 2016	1.35 (1.32–1.39)	1.22 (1.2–1.25)	1.28 (1.26–1.3)	0.95 (0.89–1.01)	0.91 (0.86–0.96)	0.92 (0.89–1.02)
Year 2017	1.44 (1.41–1.48)	1.29 (1.26–1.31)	1.35 (1.33–1.37)	0.97 (0.92–1.04)	0.95 (0.9–1)	0.96 (0.92–1.03)
Year 2018	1.47 (1.44–1.5)	1.31 (1.28–1.33)	1.37 (1.35–1.39)	0.97 (0.92–1.03)	0.98 (0.93–1.03)	0.97 (0.94–1.00)
Year 2019	1.51 (1.48–1.55)	1.37 (1.34–1.39)	1.43 (1.41–1.45)	0.98 (0.93–1.04)	0.96 (0.91–1.01)	0.97 (0.93–1.01)
Year 2020	1.65 (1.61–1.69)	1.45 (1.42–1.48)	1.53 (1.51–1.55)	1.06 (1–1.13)	1.03 (0.98–1.09)	1.05 (1.01–1.11)
Age, 60–69 years	1	1	1	1	1	1
Age, 70–79 years	3.41 (3.34–3.48)	4.19 (4.08–4.31)	3.66 (3.6–3.72)	1.05 (0.99–1.11)	1.06 (0.97–1.16)	1.05 (1.01–1.09)
Age, ≥80 years	9.6 (9.42–9.78)	13.59 (13.24–13.94)	11.07 (10.9–11.24)	1.46 (1.38–1.54)	1.58 (1.46–1.72)	1.5 (1.44–1.73)
CCI	0.84 (0.84–0.85)	0.78 (0.77–0.78)	0.81 (0.81–0.81)	1.13 (1.11–1.14)	1.2 (1.19–1.22)	1.17 (1.16–1.19)
COVID-19	1.46 (1.4–1.52)	1.32 (1.27–1.37)	1.39 (1.35–1.43)	2.84 (2.62–3.07)	2.47 (2.29–2.68)	2.66 (2.51–2.74)
Women	NA	NA	1.34 (1.33–1.35)	NA	NA	0.90 (0.89–0.91)

IHM: In-hospital mortality. T2DM: Type 2 diabetes. CCI: Charlson Comorbidity Index. OR: Odds Ratio. CI: Confidence interval.

## Data Availability

According to the contract signed with the Spanish Ministry of Health and Social Services, which provided access to the databases from the Spanish National Hospital Database (RAE-CMBD, *Registro de Actividad de Atención Especializada. Conjunto Mínimo Básico de Datos*, Registry of Specialized Health Care Activities. Minimum Basic Data Set), we cannot share the databases with any other investigator, and we have to destroy the databases once the investigation has concluded. Consequently, we cannot upload the databases to any public repository. However, any investigator can apply for access to the databases by filling out the questionnaire available at https://www.sanidad.gob.es/estadEstudios/estadisticas/estadisticas/estMinisterio/SolicitudCMBD.htm (accessed on 19 January 2023). All other relevant data are included in the paper.

## Supplementary Materials

The following supporting information can be downloaded at: https://www.mdpi.com/article/10.3390/ijerph20064923/s1, Table S1: Diagnosis analyzed with their corresponding ICD-9-CM and ICD10 codes; Table S2: Multivariate analysis of the factors associated with the presence of Alzheimer’s disease among men and women hospitalized with type 2 diabetes and factors associated with in-hospital mortality among patients with type 2 diabetes and concomitant Alzheimer’s disease, Spain, 2011–2020. Table S3: Multivariate analysis of the factors associated with the presence of vascular dementia among men and women hospitalized with type 2 diabetes and factors associated with in-hospital mortality among patients with type 2 diabetes and concomitant vascular dementia, Spain, 2011–2020.
