# Supplementary material for: Prevalence of Dementia among Patients Hospitalized with Type 2 Diabetes Mellitus in Spain, 2011–2020: Sex-Related Disparities and Impact of the COVID-19 Pandemic
Source: Int J Environ Res Public Health. 2023 Mar 10;20(6):4923. doi: 10.3390/ijerph20064923 (PMC10049429; doi:10.3390/ijerph20064923)
Supplement: Supplementary file 1 [file ijerph-20-04923-s001.zip › ijerph-2241084-supplementary.pdf]

Table S1. Diagnosis analyzed with their corresponding ICD-9-CM and ICD10 codes.

DIAGNOSIS	ICD-9-CM codes	ICD-10 codes
Type 2 diabetes	250.x0; 250.x2	E11.x
All-cause dementia	290.x, 294.1, 294.2, 294.8, 331.0–331.1, 331.82	G30.x, G31.0, G31.83, F00, F01, F02, F03, F05.1
Alzheimer dementia	331.0	G30.0, G30.1, G30.8, G30.9
Vascular dementia	290.40, 290.41	F01.50, F01.51
COVID	NA	B34.2, B97.29, U07.1

Table S2. Multivariate analysis of the factors associated with the presence of Alzheimer disease among men and women hospitalized with type 2 diabetes and factors associated with in-hospital mortality among patients with type 2 diabetes and concomitant Alzheimer disease, Spain 2011-2020.

	**Presence Alzheimer disease**			**IHM among patients with T2DM and Alzheimer disease**		
	**Men**	**Women**	**Both Sexes**	**Men**	**Women**	**Both Sexes**
	**OR(95%CI)**	**OR(95%CI)**	**OR(95%CI)**	**OR(95%CI)**	**OR(95%CI)**	**OR(95%CI)**
Year 2011	1	1	1	1	1	1
Year 2012	0.99(0.95-1.03)	1.06(1.03-1.1)	1.04(1.01-1.06)	0.94(0.84-1.05)	1.04(0.95-1.13)	1(0.94-1.08)
Year 2013	0.99(0.95-1.03)	1.01(0.97-1.04)	1(0.98-1.03)	0.96(0.86-1.08)	0.92(0.84-1)	0.93(0.87-1)
Year 2014	1.03(0.99-1.07)	1.05(1.02-1.08)	1.04(1.02-1.07)	0.92(0.82-1.03)	0.93(0.86-1.02)	0.93(0.87-1)
Year 2015	1.02(0.98-1.06)	1.07(1.04-1.1)	1.05(1.03-1.08)	1.03(0.92-1.15)	1.02(0.94-1.11)	1.02(0.96-1.09)
Year 2016	1.07(1.02-1.11)	1.14(1.11-1.18)	1.12(1.09-1.14)	0.93(0.83-1.04)	0.95(0.87-1.04)	0.94(0.88-1.01)
Year 2017	1.11(1.06-1.15)	1.16(1.13-1.2)	1.14(1.12-1.17)	0.94(0.85-1.05)	1.03(0.95-1.12)	1(0.94-1.07)
Year 2018	1.14(1.09-1.18)	1.17(1.14-1.21)	1.16(1.13-1.19)	0.99(0.89-1.09)	1.05(0.97-1.14)	1.03(0.96-1.1)
Year 2019	1.13(1.09-1.17)	1.2(1.17-1.23)	1.17(1.15-1.2)	1(0.9-1.11)	1.02(0.94-1.11)	1.02(0.95-1.08)
Year 2020	1.14(1.09-1.18)	1.23(1.19-1.26)	1.19(1.17-1.22)	1.1(0.99-1.23)	1.11(1.03-1.21)	1.11(1.04-1.19)
Age, 60-69 years	1	1	1	1	1	1
Age, 70-79 years	5.78(5.52-6.05)	6.17(5.87-6.48)	5.96(5.76-6.17)	1.25(1.08-1.45)	1.17(0.99-1.38)	1.22(1.09-1.36)
Age, ≥80 years	17.97(17.2-18.79)	18.92(18.02-19.86)	18.39(17.8-19)	1.86(1.61-2.15)	1.6(1.36-1.88)	1.74(1.56-1.94)
CCI	0.66(0.66-0.67)	0.65(0.65-0.66)	0.66(0.65-0.66)	1.1(1.07-1.12)	1.2(1.17-1.22)	1.15(1.14-1.17)
COVID	1.16(1.07-1.25)	1.26(1.18-1.33)	1.22(1.16-1.28)	3.12(2.65-3.66)	2.67(2.35-3.03)	2.83(2.56-3.12)
Women	NA	NA	1.6(1.58-1.62)	NA	NA	0.89(0.86-0.91)

IHM: In-Hospital Mortality. T2DM: Type 2 diabetes. CCI: Charlson Comorbidity Index. OR: Odds Ratio.CI: Confidence interval.

Table S3. Multivariate analysis of the factors associated with the presence of vascular dementia among men and women hospitalized with type 2 diabetes and factors associated with in-hospital mortality among patients with type 2 diabetes and concomitant vascular dementia, Spain 2011-2020.

	**Presence of vascular dementia**			**IHM among patients with T2DM and vascular dementia**		
	**Men**	**Women**	**Both Sexes**	**Men**	**Women**	**Both Sexes**
	**OR(95%CI)**	**OR(95%CI)**	**OR(95%CI)**	**OR(95%CI)**	**OR(95%CI)**	**OR(95%CI)**
Year 2011	1	1	1	1	1	1
Year 2012	1.02(0.98-1.07)	0.96(0.92-1.01)	0.99(0.96-1.02)	0.92(0.81-1.05)	1.05(0.93-1.19)	0.99(0.9-1.08)
Year 2013	0.94(0.9-0.99)	0.95(0.91-1)	0.95(0.92-0.98)	0.91(0.79-1.04)	0.98(0.86-1.11)	0.94(0.86-1.04)
Year 2014	0.95(0.91-1)	0.92(0.88-0.97)	0.94(0.91-0.97)	0.85(0.75-0.98)	0.9(0.79-1.02)	0.88(0.8-0.96)
Year 2015	0.92(0.88-0.96)	0.9(0.86-0.94)	0.91(0.88-0.94)	0.98(0.86-1.12)	0.99(0.87-1.13)	0.99(0.9-1.08)
Year 2016	0.9(0.86-0.94)	0.92(0.88-0.96)	0.91(0.88-0.94)	0.92(0.81-1.06)	0.96(0.84-1.09)	0.94(0.86-1.03)
Year 2017	0.93(0.89-0.97)	0.94(0.9-0.98)	0.93(0.91-0.96)	0.97(0.85-1.1)	1(0.88-1.13)	0.99(0.9-1.08)
Year 2018	0.9(0.86-0.94)	0.89(0.86-0.93)	0.9(0.87-0.93)	0.92(0.81-1.04)	1.15(1.02-1.3)	1.03(0.95-1.13)
Year 2019	0.95(0.91-1)	0.97(0.93-1.02)	0.96(0.94-1)	0.9(0.79-1.02)	0.99(0.88-1.12)	0.94(0.86-1.03)
Year 2020	0.96(0.92-1.01)	0.94(0.9-0.99)	0.95(0.92-0.99)	0.97(0.85-1.1)	1.04(0.91-1.18)	1(0.92-1.1)
Age, 60-69 years	1	1	1	1	1	1
Age, 70-79 years	3.09(2.97-3.22)	3.94(3.69-4.2)	3.29(3.18-3.4)	1.3(1.14-1.49)	1.23(0.99-1.54)	1.27(1.13-1.43)
Age, ≥80 years	7.08(6.81-7.36)	10.33(9.71-10.99)	8.03(7.77-8.3)	1.77(1.56-2.01)	1.78(1.44-2.2)	1.78(1.59-1.98)
CCI	1.07(1.06-1.08)	1.14(1.13-1.15)	1.1(1.1-1.11)	1.08(1.06-1.11)	1.19(1.16-1.22)	1.13(1.11-1.15)
COVID	1.17(1.06-1.29)	1.24(1.12-1.37)	1.2(1.12-1.29)	3.34(2.7-4.13)	2.9(2.33-3.6)	3.12(2.68-3.63)
Women	NA	NA	1.12(1.11-1.14)	NA	NA	0.95(0.91-0.99)

IHM: In-Hospital Mortality. T2DM: Type 2 diabetes. CCI: Charlson Comorbidity Index. OR: Odds Ratio.CI: Confidence interval.
